# No Association between Estrogen Receptor-Β Rs4986938 and Cancer Risk: A Systematic Review and Meta-Analysis

**Published:** 2019-05

**Authors:** Zhaofang LI, Xiaoli YANG, Rongqiang ZHANG, Dandan ZHANG, Baorong LI, Di ZHANG, Qiang LI, Yongmin XIONG

**Affiliations:** Institute of Endemic Diseases and Key Laboratory of Trace Elements and Endemic Diseases, National Health Commission of the People’s Republic of China, School of Public Health, Xi’an Jiaotong University Health Science Center, Xi’an, Shaanxi 710061, P.R. China

**Keywords:** ESR2, Single nucleotide polymorphism, Cancer risk

## Abstract

**Background:**

The association between estrogen receptor-β (ESR2) rs4986938 polymorphism and the risk of various types of cancer have been investigated in previous studies. However, the results remained disputable. Here, we conducted a meta-analysis to investigate the association between ESR2 rs4986938 polymorphism and the risk of cancer.

**Methods:**

We searched for relevant articles collected by the PubMed, EMBASE, and Cochrane library up to March 30, 2018. The association was assessed using Odds ratios (ORs) and 95% confidence intervals (CIs).

**Results:**

The meta-analysis involved a total of 23 studies in 20 papers, including 24,334 cases and 31,707 controls. No significant association was detected between the rs4986938 polymorphism and cancer risk in the additive model (A compared with G: OR=0.97, 95% CI=0.92–1.02, *P*=0.20), dominant model (AA+AG compared with GG: OR=0.96, 95% CI=0.93–1.03, *P*=1.00), recessive model (AA compared with AG + GG: OR=0.94, 95% CI=0.86–1.03, *P*=0.18), heterozygous model (AG compared with GG: OR=0.97, 95% CI=0.94–1.01, *P*=0.14), and homozygous model (AA compared with GG: OR=0.96, 95% CI=0.87–1.06, *P*=0.39). Results of subgroup analysis stratified by ethnicity and cancer types further validated the results.

**Conclusion:**

We found no evidence of an association between rs4986938 and the risk of overall cancer.

## Introduction

Estrogen receptors (ER), one of the family of nuclear transcription factors, are responsible for mediating the effects of steroids on many necessary functions such as cellular homeostasis, proliferation, development, reproduction, and gene expression (1, 2). ER genes including ER-α and ER-β are encoded by genes that are found on two different chromosomes: ESR1 located on chromosome 6q25.1 (3) and ESR2 located on 14q23.2 (4).

Genetic variation of the ESR genes could potentially lead to ESRs with altered binding kinetics that can adversely affect cellular metabolism (5). RNA stability of the ESR2 transcript is also explored to be affected by ESR2 rs4986938 polymorphism located in the 3’untranslated region of the gene (6). As one of the most common form of genetic variation in ESR2, rs4986938 polymorphism has been investigated in numerous studies to evaluate the association with cancer risk in multiple cancers. However, the results remained controversial.

An earlier meta-analysis reported that ESR2 rs4986938 was associated with the risk of breast cancer (BC) (7). ESR2 rs4986938 polymorphism was not significantly associated with prostate cancer (PCA) risk, either by allelic or genotypic frequencies (8). A research from Japan discovered that ESR2 rs4986938 were associated with significantly decreased risk of PCA (9). In addition, no significant differences in genotype frequencies for ESR2 rs4986938 were observed between endometrial cancer cases and controls (5). More recently, several new studies have also reported an association between ESR2 rs4986938 and cancer risk (9–11).

Owing to the inconsistent and inconclusive results found in the literature, the aim of the present meta-analysis was to provide exhaustive evidence to evaluate the effect of ESR2 rs4986938 on cancer risk. The subgroup analysis regarding ethnicity and cancer type were conducted to further analyze.

## Methods

### Literature search

We searched the PubMed, EMBASE, Cochrane library databases for relevant articles up to March 30, 2018, with the following terms: (“variants” or “polymorphisms” or “genetic polymorphism” or “single nucleotide polymorphism” or “SNP”) and (“estrogen receptors beta” or “estrogen receptor 2” or “ERbetacx” or “ESR2”) and (“tumors” or “neoplasm” or “cancer” or “carcinoma”). We had no limitations in language. Articles derived from these searches and related references cited in these articles were also reviewed.

### Inclusion/exclusion criteria

The inclusion criteria of eligible studies were as follows: (a) prospective cohort study or case-control study; (b) the studies assessed the association between ESR2 rs4986938 and cancer risk; (c) detailed genotyping data were provided; (d) cancer cases were histologically diagnosed and confirmed.

The exclusion criteria of eligible studies were as follows: (a) duplicate studies; (b) studies with insufficient genotyping data; (c) studies include case-only; (d) not related to ESR2 rs4986938 polymorphisms and cancer risk.

### Data extraction

Two reviewers (Zhaofang Li and Xiaoli Yang) independently extracted data and reached consensus regarding all the items. If controversy appeared, the third researcher (Rongqiang Zhang) participated in the discussion to resolve the issue. The extracted data included the first author, publication year, country, ethnicity, cancer type, genotyping method, source of controls, sample size, *P* value for HWE and genotype distributions in cases and controls.

### Assessment of methodology quality

The quality of the selected studies was accessed independently according to the Newcastle-Ottawa Scale (NOS). The quality score of the assessment scale was calculated by group selection, comparability and evaluation of exposure or outcome. The scores ranged from 0 to 10 and those with scores ≥6 were considered “high-quality” studies. Any discrepancies in the evaluation were settled by the third researcher (Rongqiang Zhang).

### Statistical analysis

The strength of associations between SNPs rs4986938 in ESR2 and cancer risk was analyzed by odds ratios (ORs) with 95% confidence intervals (CIs) in additive (A vs. G), dominant (AA+AG vs. GG), recessive (AA vs. AG+GG), heterozygous (AG vs GG) and homozygous (AA vs GG) models. Heterogeneity analysis was conducted using the Cochran’s Q test and I2 statistics. In any case *P*<0.10 was considered with significant heterogeneity. A random-effects model was applied when the heterogeneity was significant; otherwise, fixed-effect model was selected. Sensitivity analysis was conducted to evaluate the reliability and stability of the results by omitting one study at a time and calculating the effect size. Publication bias was accessed by the funnel plots and further performed by Egger’s test and Begg’s test. All tests carried out in the present report were two-tailed and *P* ≤ 0.05 was considered to be statistically significant. Data were performed using the Stata software (version 12.0; StataCorp LP, College Station, TX, USA) and RevMan software (version 5.3; The Nordic Cochrane Centre, Copenhagen, Denmark).

## Results

### Study selection and characteristics

A total of 210 publications were identified through the literature search. After removing the duplicate articles, 178 articles are still available for subsequent evaluation.

**Fig. 1:**
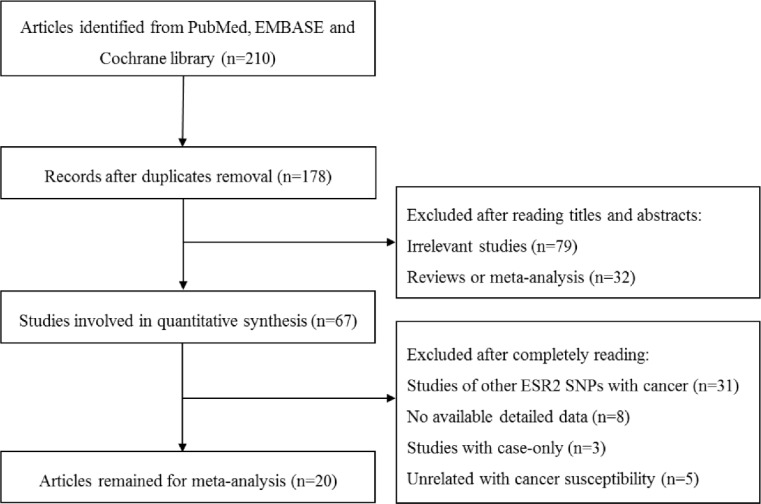
The flow diagram of identification for studies included

Another 111 articles containing 32 reviews and/or meta-analysis and 79 irrelevant articles were excluded after screening the titles and abstracts. Finally, 20 articles were included in the present study after reading in greater detail (Fig. 1). A total of 23 studies from 20 papers including 24,334 cases and 31,707 controls met the inclusion criteria in the meta-analysis, 2 were cohort studies and the other 21 studies were case-control studies. The rs4986938 polymorphisms were in HWE for all studies. Among the 23 studies, 8 were conducted in USA (8, 12–16), 3 in Japan (9, 17), 2 in Sweden (6, 18) and Germany (19, 20), and 1 each in Tunisia (10), Brazil (11), Singapore (21), Iran (22), China (23), Australia (5), France (24) and India (25).

The cancer types analyzed in these studies were breast cancer (BC); prostate cancer (PCA); lung cancer (LC); colorectal adenoma (CRA); biliary tract cancers (BTC); and endometrial cancer (EC). The characteristics of the eligible studies are summarized in Tables 1 and 2.

**Table 1:** Characteristics of the eligible studies

***Study***	***Year***	***Country***	***Ethnicity***	***Cancer type***	***Genotyping method***	***Control Source***	***case/control***	***Quality score***
Ghali, R.M.	2018	Tunisia	African	BC	TaqMan	HB+PB	207/284	H
Rezende, L.M.	2017	Brazil	Caucasian	BC	RFLP-PCR	NA	253/257	H
Lu, X.	2015	Japan	Asian	PCA	TaqMan	HB	352/352	L
Lim, W.	2012	Singapore	Asian	LC	TaqMan	HB	559/988	H
Safarinejad, M.R.	2012	Iran	Asian	PCA	PCR-RFLP	PB	162/324	H
Levine, A.J.	2012	American	Mixed race	CRA	Illumina’s bead array	HB	655/696	L
Paulus, J.K.	2011	American	Mixed race	LC	TaqMan	HB+PB	1021/826	H
Sainz.	2011	Germany	Caucasian	CRC	PCR-ARMS	HB	1752/1774	H
Su, M.C.G.	2010	Germany	Caucasian	BC	Mass ARRAY	PB	3149/5489	H
Park, S.K.	2010	China	Asian	BTC	TaqMan	PB	411/786	H
Ashton, K.A.	2009	Australia	Caucasian	EC	RFLP-PCR	PB	191/291	H
Iwasaki, M.1.	2009	Japan	Asian	BC	TaqMan	HB	388/388	H
Iwasaki, M.2.	2009	Japan	Caucasian	BC	TaqMan	HB	458/458	H
Nicolaiew.	2009	France	Caucasian	PCA	DHPLC	HB	286/285	L
Chae, Y.K.	2009	American	Caucasian	PCA	TaqMan	PB	269/440	H
Surekha, D.	2009	India	Asian	BC	RFLP-PCR	HB	250/250	L
Cox, D.G.	2008	American	Caucasian	BC	TaqMan	PB	5789/7761	H
Chen.1.	2007	American	African	PCA	TaqMan	PB	773/961	H
Chen.2.	2007	American	Asian	PCA	TaqMan	PB	459/471	H
Chen.3.	2007	USA and Europe	Caucasian	PCA	TaqMan	PB	5917/6551	H
Gallicchio, L.	2006	American	Caucasian	BC	TaqMan	PB	91/1347	H
Maguire, P.	2005	Sweden	Caucasian	BC	Pyrosequencing	HB	723/480	L
Forsti, A.	2003	Sweden	Caucasian	BC	RFLP-PCR	PB	219/248	H

BC: breast cancer; PCA: prostate cancer; LC: lung cancer; CRA: colorectal adenoma; BTC: biliary tract cancers; EC: endometrial cancer

**Table 2:** ESR2 rs4986938 polymorphism genotype distribution and allele frequency in cases and controls

***Study***	***Year***	***Case***				***Control***			
		***Total***	***GG***	***AG***	***AA***	***Total***	***GG***	***AG***	***AA***
Ghali, R.M.	2018	201	55	99	47	283	94	120	69
Rezende, L.M.	2017	257	97	129	31	253	109	115	29
Lu, X.	2015	352	280	67	5	352	254	90	8
Lim, W.	2012	544	446	95	3	964	807	148	9
Safarinejad, M.R.	2012	162	81	76	5	324	159	124	41
Levine, A.J.	2012	648	322	257	69	683	311	294	78
Paulus, J.K	2011	1021	378	485	155	826	303	394	129
Sainz.	2011	1752	665	825	262	1774	695	815	264
Su, M.C.G.	2010	3140	1277	1431	432	5478	2169	2557	752
Park, S.K.	2010	302	11	94	197	772	13	170	589
Ashton, K.A.	2009	188	87	78	23	286	116	128	42
Iwasaki, M.1.	2009	388	289	94	5	388	281	102	5
Iwasaki, M.2.	2009	458	228	180	50	458	236	171	51
Nicolaiew.	2009	286	138	100	48	285	122	116	47
Chae, Y.K.	2009	219	81	105	33	370	134	185	51
Surekha, D.	2009	248	21	95	132	249	9	81	159
Cox, D.G.	2008	5600	2513	2382	705	7517	3229	3304	984
Chen.1.	2007	773	408	300	65	961	538	360	63
Chen.2.	2007	459	315	131	13	471	346	122	3
Chen.3.	2007	5917	2274	2739	904	6551	2481	3039	1031
Gallicchio, L.	2006	88	26	43	19	1272	470	612	190
Maguire, P.	2005	696	298	315	83	421	175	190	56
Forsti, A.	2003	219	95	99	25	238	105	103	30

PCR: polymerase chain reaction; RFLP: restriction fragment length polymorphism; PB: population based; HB: hospital-based; H: high-quality; L: low-quality; HWE: Hardy-Weinberg equilibrium; HWE (*P*) = >0.05

### Association between rs4986938 in ESR2 and cancer risk

**Fig. 2:**
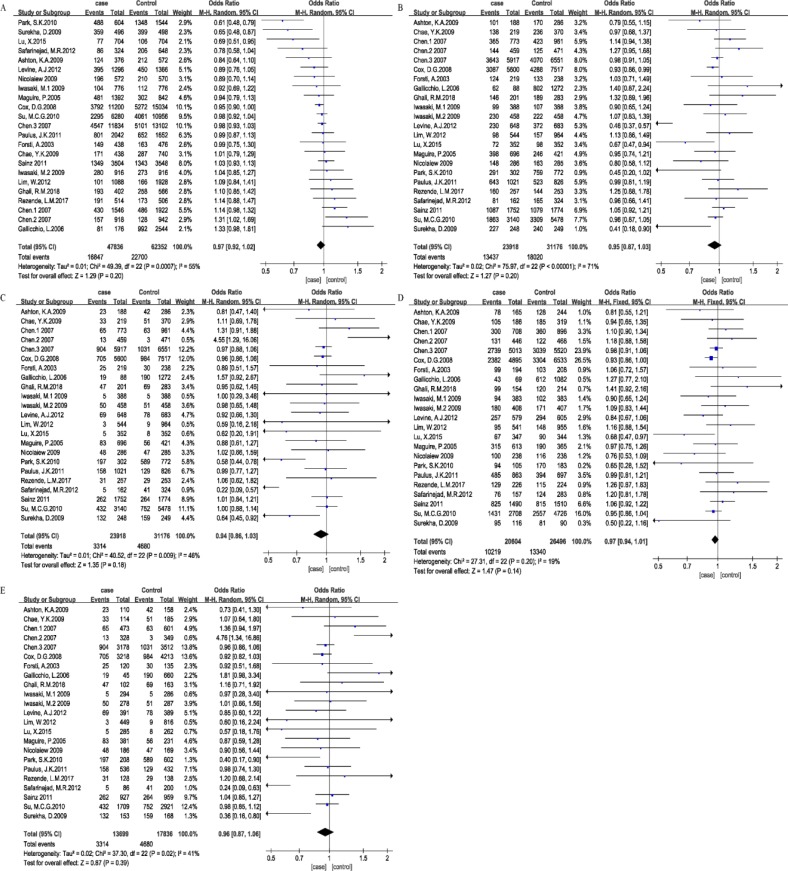
Forest plot of cancer risk associated with rs4986938. Note: (A) Allelic model, (B) dominant model, (C) recessive model, (D) heterozygous model, (E) homozygous model

No significant association was detected between the rs4986938 polymorphism and cancer risk in the additive model (A compared with G: OR=0.97, 95% CI=0.92–1.02, *P*=0.20), dominant model (AA+AG compared with GG: OR=0.96, 95% CI=0.93–1.03, *P*=1.00), recessive model (AA compared with AG + GG: OR=0.94, 95% CI=0.86–1.03, *P*=0.18), heterozygous model (AG compared with GG: OR=0.97, 95% CI=0.94–1.01, *P*=0.14), and homozygous model (AA compared with GG: OR=0.96, 95% CI=0.87–1.06, *P*=0.39, Table 3). The Forest plot of cancer risk associated with rs4986938 was shown in Fig. 2.

**Table 3:** Meta-analysis of the association between rs4986938 polymorphism and cancer risk. OR: odds ratio; CI: confidence intervals; N: number of included studies; R: random-effect model; F: fixed-effect method

***Genetic models***	***N***	***Test of association***		***Model***	***Test of heterogeneity***		***(Egger) P-value***
		***OR (95%CI)***	***P-value***		***P-value***	***I2 (%)***	
Allelic model (A vs. G)	23	0.97 (0.92,1.02)	0.20	R	0.0007	55	0.746
Caucasian	13	0.97 (0.95,1.00)	0.07	F	0.49	0	
Asian	8	0.85 (0.69,1.04)	0.11	R	0.0003	74	
African	3	1.13 (0.99,1.28)	0.07	F	0.75	0	
Breast cancer	10	0.97 (0.93,1.00)	0.06	F	0.09	40	
Prostate cancer	7	1.00 (0.91,1.11)	0.94	R	0.01	63	
Lung cancer	2	1.01 (0.90,1.14)	0.88	F	0.53	0	
Dominant model (AA+AG vs GG)	23	0.96 (0.93,1.03)	1.00	R	<0.000001	71	0.729
Caucasian	13	0.95 (0.90,1.00)	0.04	F	0.47	0	
Asian	8	0.88 (0.70,1.10)	0.26	R	0.02	59	
African	3	1.16 (0.98,1.38)	0.08	F	0.60	0	
Breast cancer	10	0.95 (0.91,1.00)	0.07	F	0.14	33	
Prostate cancer	7	1.00 (0.95,1.06)	0.92	F	0.10	44	
Lung cancer	2	1.03 (0.88,1.20)	0.72	F	0.43	0	
Recessive model (AA vs AG+GG)	23	0.94 (0.86,1.03)	0.18	R	0.009	46	0.597
Caucasian	13	0.98 (0.92,1.03)	0.41	F	0.95	0	
Asian	8	0.70 (0.46,1.05)	0.09	R	0.02	56	
African	3	1.14 (0.87,1.50)	0.34	F	0.25	23	
Breast cancer	10	0.96 (0.89,1.03)	0.29	F	0.45	0	
Prostate cancer	7	1.00 (0.79,1.28)	0.97	R	0.005	68	
Lung cancer	2	0.97 (0.76,1.24)	0.80	F	0.45	0	
Heterozygote model (AG vs GG)	23	0.97 (0.94,1.01)	0.14	F	0.20	19	0.662
Caucasian	13	0.96 (0.93,1.01)	0.09	F	0.58	0	
Asian	8	0.98 (0.85,1.12)	0.77	F	0.10	41	
African	3	1.14 (0.95,1.37)	0.15	F	0.44	0	
Breast cancer	10	0.96 (0.91,1.01)	0.10	F	0.29	17	
Prostate cancer	7	1.01 (0.95,1.07)	0.83	F	0.21	28	
Lung cancer	2	1.04 (0.88,1.23)	0.62	F	0.36	0	
Homozygote model (AA vs GG)	23	0.96 (0.87,1.06)	0.39	R	0.02	41	0.627
Caucasian	13	0.96 (0.90,1.02)	0.18	F	0.81	0	
Asian	8	0.65 (0.35,1.20)	0.17	R	0.01	61	
African	3	1.29 (0.96,1.73)	0.10	F	0.62	0	
Breast cancer	10	0.95 (0.88,1.03)	0.20	F	0.22	24	
Prostate cancer	7	1.01 (0.79,1.31)	0.92	R	0.005	68	
Lung cancer	2	0.96 (0.73,1.26)	0.76	F	0.48	0	

### Subgroup analysis

Due to the existence of heterogeneity, analysis of stratification was performed based on ethnicity and cancer type. In the subgroup analysis based on ethnicity, 13 Caucasian studies, 8 Asian studies and 3 African studies found no significant association between rs4986938 in ESR2 and cancer risk in any genetic model (Table 3).

**Fig. 3:**
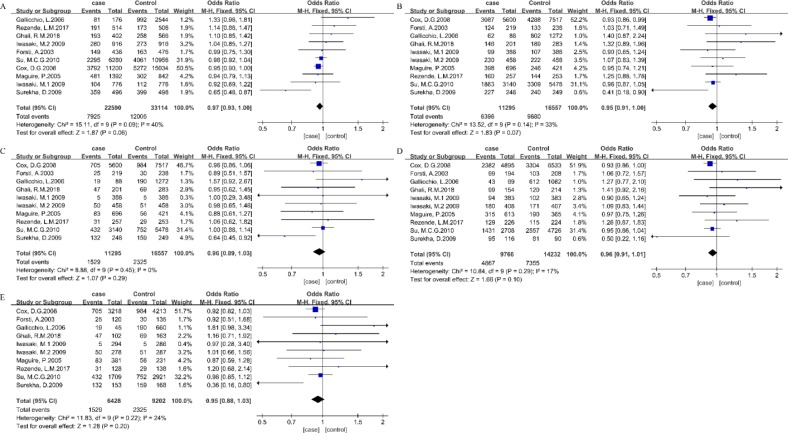
Forest plots of ORs for the association between ESR2 rs4986938 and BC. Note: (A) Allelic model, (B) dominant model, (C) recessive model, (D) heterozygous model, (E) homozygous model

**Fig. 4:**
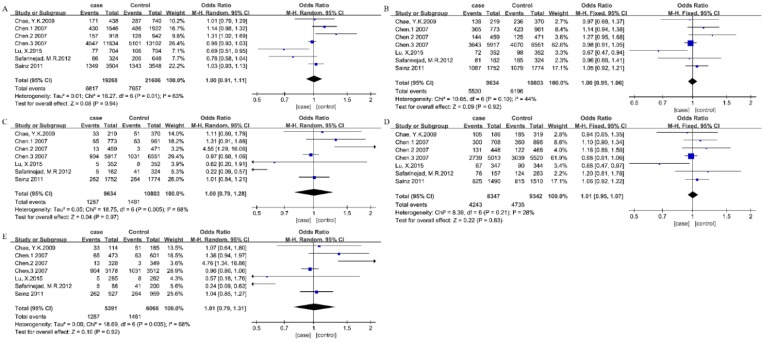
Forest plots of ORs for the association between ESR2 rs4986938 and PCA. Note: (A) Allelic model, (B) dominant model, (C) recessive model, (D) heterozygous model, (E) homozygous model

In the stratified analysis by cancer type, 10 studies were used to evaluate the relationship between ESR2 rs4986938 polymorphism and BC risk. No significant association was detected between the rs4986938 polymorphism and breast cancer risk in any genetic model (Fig. 3, Table 3). Meanwhile, no significant association was detected between the rs4986938 polymorphism and PCA risk in any genetic model (Fig. 4, Table 3).

### Sensitivity analysis and publication bias

**Fig. 5:**
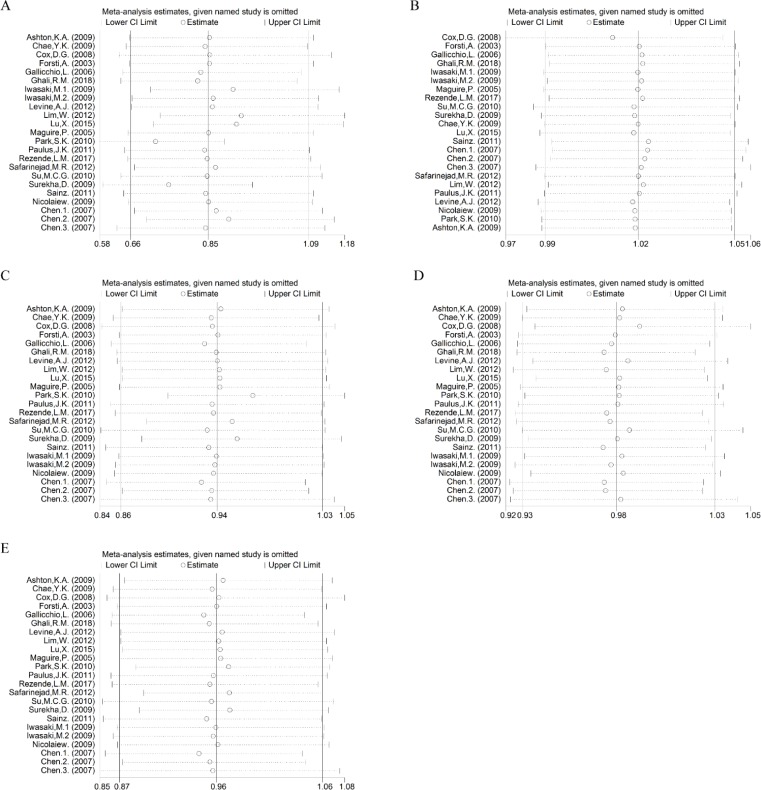
Sensitivity analyses of ESR2 rs4986938 in five genetic models. Note: (A) Allelic model, (B) dominant model, (C) recessive model, (D) heterozygous model, (E) homozygous model

Sensitivity analysis was performed to explore the influence of a single study on the overall risk estimated by removing one study at a time. The ORs were not altered significantly (Fig. 5).

**Fig. 6:**
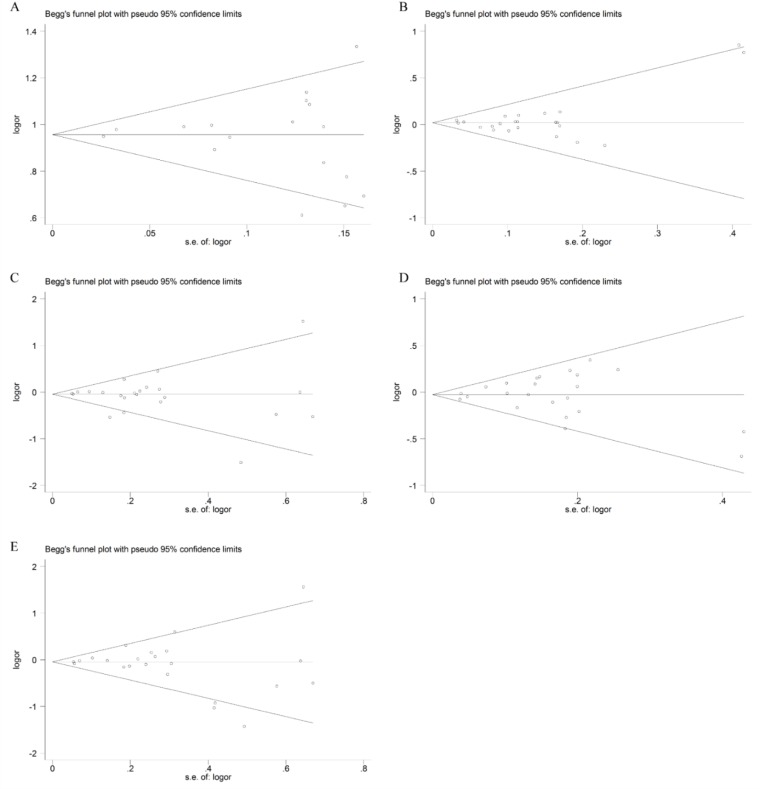
Results of Begg’s tests in five genetic models. Note: (A) Allelic model, (B) dominant model, (C) recessive model, (D) heterozygous model, (E) homozygous model

Begg’s and Egger’s tests were conducted to evaluate the publication bias. The shape of the funnel plot did not reveal any obvious asymmetry (Fig. 6). The *P* values for the Egger’s test are shown in Table 3.

## Discussion

Estrogens could influence many physiological processes in mammals including reproduction, cardiovascular health, bone integrity, cognition, and behavior (26). In many diseases, estrogen mediates its effects through the estrogen receptor (ER), which serves as the basis for many therapeutic interventions (26). Rs4986938 of ESR2 has been investigated in many types of cancer. In the present meta-analysis, we systematically analyzed the association between ESR2 rs4986938 and cancer risk. Our results showed that there was no association between ESR2 rs4986938 and cancer risk in all genetic models. In the subgroup analysis based on ethnicity, results showed that Caucasian, Asian and African studies found no significant association between rs4986938 in ESR2 and cancer risk in any genetic model. Meanwhile, no significant association was detected between the rs4986938 polymorphism and the risk of BC and PCA in any genetic model.

Previous meta-analysis studies have been conducted to elucidate the association between the rs4986938 polymorphism and the risk of cancer. In a previous meta-analysis (7), including 22833 cases and 30319 controls, ESR2 rs4986938 was likely to be related to breast cancer risk, and only contained one type of tumor. In another meta-analysis (27), including 22833 cases and 30319 controls, results showed that ESR2 rs4986938 polymorphism was associated with decreased breast cancer and ethnicity subgroup analysis observed a decreased risk in both Asian and Caucasian descendent. Owing to the inconsistent and inconclusive results found in previous meta-analysis, the need for additional studies examining the effect of ESR2 rs4986938 on cancer risk seems of vital importance. Besides, our analysis included relevant studies published during the transition period since the previous meta-analysis were carried out. This may be the reason for the inconsistent results. Moreover, we included Africans in our meta-analysis with BC to discover the association between ESR2 rs4986938 and BC which other meta-analysis didn’t. To the best of our knowledge, this is the largest and most comprehensive meta-analysis of 23 studies including 24,334 cases and 31,707 controls to determine the association between ESR2 rs4986938 and risk of cancer.

To determine the influence of population stratification, all the data were divided into 3 subgroups: Caucasian, Asian and African. Results showed that polymorphism of rs4986938 had no association with cancer risk in Caucasian, Asian and African subgroup. Our combined analysis was in line with Xia’s (28) analysis that no significant association was detected between the rs4986938 polymorphism and cancer risk. However, due to the existence of heterogeneity, the negative result of the association should be interpreted carefully. Besides, larger sample sizes of studies are needed to confirm the results.

BC is the leading cancer in females worldwide, and the second cause of death among women (28). In the subgroup meta-analysis under cancer types, no significant association was found between ESR2 rs4986938 variant and BC. Our conclusion was different from another study that concluded SNP rs4986938 might be associated with BC (7). The present meta-analysis contained 2 updated literatures which coincide with our conclusion (10, 11). Besides, 6 studies published previously also observed no significant association between these gene polymorphisms and susceptibility to BC ( 10, 11, 14, 29–31). It is likely that other genetic and environmental factors had influenced BC development (32).

As regarded to the other cancers, no significant association was found between rs4986938 and PCA. ESR2 is regulated by AR and interacts with ESR1 to regulate prostate carcinogenesis through the modulation of genes involved in cell proliferation and apoptosis (16). However, the associations between ESR2 rs4986938 and PCA have been inconclusive. We pooled the data of 7 studies containing 9634 cases and 10803 controls to clarify the association of ESR2 rs4986938 and prostate cancer. The previous meta-analyses also support our findings (26).

Several limitations in our study should be mentioned. First, owning to the small sample of African data, the effects of rs4986938 on African populations need to be investigated in large scale and well-designed studies. In addition, the researches about the association of rs4986938 polymorphisms with other cancers are still a relatively emerging field which made it impossible to perform subgroup analysis. Lastly, as positive results are more likely to be published than negative results, it was unavoidable that publication bias lead to the overestimation of effects in meta-analyses.

## Conclusion

We systematically reviewed the relationship between rs4986938 polymorphisms and overall cancer risk. We found no evidence of an association between rs4986938 and the risk of overall cancer.

## Ethical considerations

Ethical issues (Including plagiarism, informed consent, misconduct, data fabrication and/or falsification, double publication and/or submission, redundancy, etc.) have been completely observed by the authors.
